# The Protein Interaction of RNA Helicase B (RhlB) and Polynucleotide Phosphorylase (PNPase) Contributes to the Homeostatic Control of Cysteine in Escherichia coli*

**DOI:** 10.1074/jbc.M115.691881

**Published:** 2015-10-20

**Authors:** Yi-Ting Tseng, Ni-Ting Chiou, Rajinikanth Gogiraju, Sue Lin-Chao

**Affiliations:** From the ‡ Institute of Molecular Biology, Academia Sinica, Taipei 11529, Taiwan,; the § Institute of Molecular Medicine, College of Medicine, National Taiwan University, Taipei 10617, Taiwan,; the ¶ Institute of Biochemistry & Molecular Biology, National Yang-Ming University, Taipei 11221, Taiwan

**Keywords:** bacterial metabolism, Escherichia coli (E. coli), exosome complex, oxidative stress, ribonuclease, RNA degradation, Cysteine homeostasis, PNPase, RhlB

## Abstract

PNPase, one of the major enzymes with 3′ to 5′ single-stranded RNA degradation and processing activities, can interact with the RNA helicase RhlB independently of RNA degradosome formation in *Escherichia coli*. Here, we report that loss of interaction between RhlB and PNPase impacts cysteine homeostasis in *E. coli*. By random mutagenesis, we identified a mutant RhlB^P238L^ that loses 75% of its ability to interact with PNPase but retains normal interaction with RNase E and RNA, in addition to exhibiting normal helicase activity. Applying microarray analyses to an *E. coli* strain with impaired RNA degradosome formation, we investigated the biological consequences of a weakened interaction between RhlB and PNPase. We found significant increases in 11 of 14 genes involved in cysteine biosynthesis. Subsequent Northern blot analyses showed that the up-regulated transcripts were the result of stabilization of the *cysB* transcript encoding a transcriptional activator for the *cys* operons. Furthermore, Northern blots of PNPase or RhlB mutants showed that RhlB-PNPase plays both a catalytic and structural role in regulating *cysB* degradation. Cells expressing the RhlB^P238L^ mutant exhibited an increase in intracellular cysteine and an enhanced anti-oxidative response. Collectively, this study suggests a mechanism by which bacteria use the PNPase-RhlB exosome-like complex to combat oxidative stress by modulating *cysB* mRNA degradation.

## Introduction

mRNA is unstable, and its stability is post-transcriptionally controlled to quickly change gene expression to adapt growth to new environments. In *Escherichia coli*, the RNA degradosome comprises many enzymes and minor components (1–4) that can help to respond to environmental changes by modulating mRNA stability. The major components include the endoribonuclease RNase E, the 3′ to 5′ single-stranded RNA degrading enzyme polynucleotide phosphorylase (PNPase),^3^ the DEAD box RNA helicase RhlB, and the glycolytic enzyme enolase (5–7). Although PNPase and RhlB can interact independently of RNase E (8, 9), the importance of this interaction has not been studied in depth.

The bacterial PNPase complex is a trimeric complex, and each PNPase has two RNase PH domains followed by S1 and KH domains, all of which are important for RNA binding (10). The six RNase PH domains of the trimeric complex are arranged around a central pore with a diameter of ∼14 Å—just large enough to accommodate a single single-stranded RNA molecule (11). This structural feature ensures that the bacterial PNPase complex exhibits a preference for the degradation of single-stranded RNA, and functionality ceases when the machinery arrives at a long double-stranded stem in the 3′ end of mRNA (12). Thus, *in vivo*, the degradation of RNA by PNPase probably requires cofactors such as RNA helicase to efficiently unfold RNA secondary structures.

In *E. coli*, the helicase RhlB interacts with PNPase independently of RNA degradosome formation (8, 9). *In vitro* analysis has shown that RhlB helps PNPase to degrade double-stranded RNAs (8). Moreover, the reconstitution of a minimal RNA degradosome demonstrated that RhlB enables PNPase to mediate the degradation of a repetitive extragenic palindrome sequence-containing transcript, *i.e. malEF* (13). Interestingly, eukaryotic and archaea cells have 3′ to 5′ exoribonuclease complexes with a core-exosome that is structurally similar to PNPase (14, 15). It has also been shown that the eukaryotic exosome associates with a variety of accessory factors in a cell compartment- and species-dependent manner to mediate RNA degradation and processing (16–23). It is not yet understood how a ribonuclease-protein complex selects its specific mRNA substrate and thus specifically controls degradation.

In this study, we examined the importance of the protein interaction between RhlB and PNPase for mRNA stability in the absence of the degradosome. We isolated an RhlB mutant, RhlB^P238L^, with an impaired PNPase, but not RNase E, interaction. Microarray analysis of cells bearing this mutant protein revealed altered expression profiles of cysteine regulon genes responsible for control of cysteine biosynthesis. In *E. coli*, cysteine biosynthesis is mediated by the cysteine regulon that includes 7 operons: *sbp*, *cysPUWA*, *cysM*, *cysJIH*, *cysDNC*, *cysK*, and *cysE*, containing 14 genes. With the exceptions of *sbp* and *cysM*, deficiency of any other gene of the cysteine regulon renders the cell a cysteine auxotroph (24, 25), underscoring the importance of controlled expression. Cells with reduced RhlB-PNPase interactions also have a prolonged half-life of *cysB* mRNA, a dual transcription factor (26) that activates the expression of all cysteine regulon genes except *cysE*. Further, cells expressing the RhlB^P238L^ mutant exhibit increased intracellular cysteine and increased anti-oxidative ability. These data provide a possible mechanism by which bacteria may modulate cysteine biosynthesis through the exosome-like complex to combat oxidative stress.

## Experimental Procedures

### 

#### 

##### Bacterial Strains and Plasmids

The bacterial strains and plasmids used are listed in Table 1. Strains were constructed using P1 transduction using Δ*rhlB* mutant SU02 (27) or Keio collection strain JW3582 (Δ*cysE*), JW3686 (Δ*tnaA*), or JW5808 (Δ*pncB*) as donor, as described (28). E166K mutation on RhlB (29) or N435D mutation on PNPase (30) was generated in FLAG-tagged plasmid by using QuikChange® II XL site-directed mutagenesis kits (Stratagene).

**TABLE 1:** **Strains and plasmids used in this study**

Strain	Genotype	Source
DHP1	*Cya*	Ref. 31
BL21(DE3)	λ(*DE3 [lacI lacUV5-T7 gene 1 ind1 sam7 nin5]*)	Ref. 59
BL21(DE3) Δ*rhlB*	*rhlB*::*Kan* in BL21(DE3)	This study
BL21(DE3) *rne131*	*rne131* in BL21(DE3)	Ref. 33
BL21(DE3) *rne131* Δ*rhlB*	*rhlB*::*Kan* in BL21(DE3) *rne131*	Ref. 9
BL21(DE3) *rne131* Δ*pnp*	*pnp*::*Kan* in BL21(DE3) *rne131*	This study
BL21(DE3) *rne131* Δ*rhlB* Δ*pnp*	*rhlB pnp*::*Kan* in BL21(DE3) *rne131*	This study
BL21(DE3) *rne131* Δ*pcnB*	*pcnB*::*Kan* in BL21(DE3) *rne131*	This study
BL21(DE3) *rne131* Δ*rhlB* Δ*cysE* Δ*tnaA*	*cysE tnaA*::*Kan* in BL21(DE3) *rne131* Δ*rhlB*	This study

**Plasmid**		
pT25	T25-expressed plasmid	Ref. 31
pT25RhlB^wt^	RhlB^wt^ tagged with T25	Ref. 8
pT25RhlB^P238L^	RhlB^P238L^ tagged with T25	This study
pPNPT18	PNPase tagged with T18	Ref. 8
pRE12T18	Residue 684–784 of RNase E tagged with T18	Ref. 8
pFlagRhlB^wt^	Flag-tagged wild-type RhlB	Ref. 8
pFlagRhlB^P238L^	Flag-tagged RhlB^P238L^	This study
pFlagRhlB^E166K^	Flag-tagged RhlB^E166K^	This study
pFlagPNP	Flag-tagged PNP	Ref. 8
pFlagPNP^N435D^	Flag-tagged PNP^N435D^	This study
pACYCDeut-CysE^M256I^	Cysteine-insensitive CysE mutant	This study

##### Screening of a Mutant RhlB That Reduces Interactions between RhlB and PNPase Using a Bacterial Two-hybrid System

Random mutagenesis was performed using an error prone (1–3 mutations per *rhlB* DNA fragment) PCR kit (GeneMorph® II random mutagenesis kit; Stratagene), and mutants with weakened protein interactions were identified as per the method described by Karimova *et al.* (31). In brief, *rhlB* DNA fragment PCR products resulting from the error prone PCR were digested with PstI and BamHI, followed by cloning into a pT25 plasmid that expresses a T25 fragment corresponding to amino acids 1–224 of CyaA (adenylate cyclase) as an N-terminal tag. The resulting plasmid was named pT25RhlB. Wild-type *pnp* with a T18 plasmid expressing the T18 fragment corresponding to amino acids 225–399 of CyaA as a C-terminal tag was also prepared (pPNPT18). Only tagged interacting protein partners can induce CyaA activity by bringing the N- and C-terminal regions of CyaA together. Mutated pT25RhlB pool and wild-type pPNPT18 (8) were cotransformed into a DHP1 strain (an adenylate cyclase-deficient derivative of DH1) to screen for protein-protein interactions as described (31). β-Galactosidase activity assays were performed as described previously (8) to measure the strength of interactions between mutant RhlB and PNPase *in vivo*. To exclude false positive results caused by different protein expression levels, we performed Western blotting using α-RhlB antibody to confirm the expression level of mutant T25RhlB proteins. White transformants with a similar expression protein level to wild-type T25RhlB were selected and isolated for sequencing confirmation.

##### BIAcore Surface Plasmon Resonance Analysis

Real time protein-protein interaction strength was examined by means of a Biacore instrument (Biacore X) as described (8). pFlagRhlB (8), pFlagRhlB^P238L^, and pFlagPNP (8) were used for protein purification as described previously (6). The identified mutation (P238L) was introduced into pFlagRhlB by QuikChange® II XL site-directed mutagenesis kits (Stratagene). Purified FlagPNPase was immobilized on a CM5 sensor chip using an amine-coupling kit (Amersham Biosciences). Different concentrations, ranging from 0.75 to 24 μm of purified FlagRhlB^wt^ or FlagRhlB^P238L^, were injected with a constant (10 μl/min) flow rate at 25 °C. The kinetic analysis was performed using BIAcore evaluation software.

##### Helicase Activity Assay

Helicase activity was measured as described previously (8), with minor modifications. The 5′-labeled longer strand and the unlabeled shorter strand RNA were hybridized to form duplex RNA in a 1:1 ratio. The duplex RNA was incubated with purified FlagRhlB^wt^ or FlagRhlB^P238L^ at 30 °C in final reaction volumes of 20 μl containing 20 mm Tris-HCl, pH 7.5, 5 mm MgCl_2_, 0.1 mm DTT, 100 mm NaCl, 5% glycerol, and 0.1% Triton X-100. Samples were separated on a 16% polyacrylamide (19:1 bis-acrylamide) native gel, visualized by autoradiography, and quantified by LAS-1000 plus (Fuji Film).

##### Microarray Measurements of RNA Abundance and Data Processing

BL21(DE3) *rne131* Δ*rhlB* carrying FLAG-tagged wild-type or mutant (P238L) RhlB were grown at 30 °C in LB medium to an *OD*_600_ of 0.55–0.6. To distinguish the effect of the RhlB-PNPase interaction from that of the RNA degradosome, we used an *E. coli* strain with a truncated *rne* gene (*rne131*) encoding the N-terminal domain of RNase E (residues 1–584) (32, 33). RNA was isolated according to the RNeasy® mini kit (Qiagen) manufacturer's protocol. Technical support for microarray experiments was provided by the Institute of Molecular Biology (Academia Sinica) Microarray Core Facility. The relative mRNA levels were determined by parallel two-color hybridization of DNA microarrays as described previously (34, 35). RNA samples taken from BL21(DE3) *rne131* Δ*rhlB* expressing FlagRhlB^wt^ or FlagRhlB^P238L^ were synthesized into cDNA and then labeled with Alexa Fluor® 647 (Molecular Probes, Invitrogen). Relative mRNA abundance was measured using BL21(DE3) *rne131* Δ*rhlB* cells expressing FLAG tag only as reference, and the RNA sample was then synthesized into cDNA and labeled with Alexa Fluor® 555 (Molecular Probes, Invitrogen). Synthesis of cDNA, hybridization, and analysis of spots were performed as described (35). The microarray data have been deposited at GEO database (GSE: 57784).

Assistance with data analysis was provided by the Institute of Molecular Biology Bioinformatics Core Facility. The microarray data were first subject to intensity-dependent LOWESS normalization using the “per spot and per chip” setting in the GeneSpring software (Agilent Technologies). To find the significantly expressed genes within each of the sample triplicates, we subjected gene lists to significance analysis for the microarray package, implemented in the TIGR MultiExperiment viewer (The Institute for Genomic Research, Rockville, MD). The missing values were imputed before testing using the *K* nearest neighbor method, where *K* = 6. The false discovery rates within and among sample groups were estimated by a bootstrap resampling method, and false discovery rate thresholds of 5% or less were established to obtain significantly expressed genes.

##### RNA Stability Assay

Bacteria were grown in LB medium at 30 °C to an *OD*_600_ of 0.5–0.6, and total RNA was extracted as described previously (36) at different time points after the addition of rifampicin (0.5 mg/ml). Specific RNA was detected by RNA probe labeling with digoxigenin. Briefly, 5 μg of total RNA of each strain was separated on 1.2% formaldehyde agarose gel and transferred to a Hybond N+ membrane (Amersham Biosciences) through capillary action with a stack of towels 10 centimeters high. For hybridization, RNA probes were internally labeled with digoxigenin-11-UTP and hybridized to the membrane with a digoxigenin Northern starter kit (Roche) (37). Northern blot signals were visualized using a UVP digital image system and quantified via ImageJ software 1.50b.

##### Growth Inhibition Assay

Bacteria were grown at 37 °C to an *OD*_460_ = 0.4 in Vogel-Bonner medium E (0.8 mm MgCl_2_·7H_2_O, 10.4 mm citric acid, 57.4 mm K_2_HPO_4_, 25.4 mm NaNH_4_PO_4_·4H_2_O) (38), supplemented with 0.5 mm glutathione as the organic sulfur source and 0.2% glucose as the carbon source. The culture was further divided into two flasks supplied with either 0.5 mm cysteine or an equal volume of autoclaved double distilled H_2_O. The *OD*_460_ was measured every 20 min for an hour, and growth inhibition (%) was calculated using the following formula,

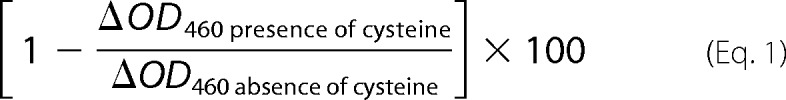
 where Δ*OD*_460_ is the difference in *A* after 1 h of incubation.

##### Anti-oxidative Stress Assay

The bacterial strain BL21(DE3) *rne131* Δ*rhlB* Δ*cysE* Δ*tnaA* was used to examine whether impaired RhlB-PNPase interactions resulted in impaired anti-oxidative resistance. To measure the effect of cysteine biosynthesis, we removed chromosomal *cysE* and induced expression of a cysteine-insensitive mutant (CysE^M256I^) under the control of its own promoter (39–41). A PCR-generated EcoNI-NdeI fragment encoding the full transcription unit and the promoter of *cysE* was cloned into pACYCDeut-1 (EMD Millipore), and the M256I mutation was introduced into pACYCDeut-CysE by QuikChange® II XL site-directed mutagenesis kits (Stratagene). To analyze the effects of weakened RhlB-PNPase interactions on cysteine synthesis, chromosomal *tnaA* was removed and replaced by a kanamycin cassette as described under “Bacterial Strains and Plasmids” above. The strains containing pFlagRhlB^wt^ or pFlagRhLB^P238L^ were grown in LB medium at 37 °C overnight. The overnight cultures were further diluted to an *OD*_600_ value of 0.1, and 2 μl of each diluted culture was spotted on LB plates with 0–1 mm H_2_O_2_ (Merck) as indicated in Fig. 8. For the oxidative stress induced by paraquat (PQ; Sigma), 2 μl of each culture was serially diluted and spotted on the plate with or without 0.4 mm PQ. The plates were incubated at 37 °C overnight, and the stress resistance was measured by observing colony formation ability.

##### Measurement of Cysteine Content from Bacterial Metabolite Extract by LC-MS/MS

Bacteria were grown at 37 °C to an *OD*_460_ = 0.4 in M9 medium (2.2 mm KH_2_PO_4_, 1.87 mm NH_4_Cl, 4.23 mm Na_2_HPO_4_, 0.86 mm NaCl) supplemented with 0.2% glucose, 1 mm MgSO_4_·7H_2_O, 0.1 mm CaCl_2_, 1 μg/ml vitamin B_1_, and 100 mg/liter each of l-isoleucine, l-leucine, l-methionine, and glycine amino acids. The amino acids were added to avoid the growth inhibition caused by increased l-cysteine (42, 43). The metabolites were extracted as described previously (44), with 21 μg/ml l-[1-C13] cysteine (Icon Services Laboratory) as an internal standard. The final extracted metabolites were lyophilized using a miVac Duo concentrator (GeneVac) and then resuspended in 700 μl of 50:50 methanol/water.

LC-MS/MS analysis was supported by the Metabolomics Core Facility of the Scientific Instrument Center (Academia Sinica). LC-MS/MS was performed on a LTQ-Orbi Elite system (Thermo Scientific) equipped with an ACQUITY ultra performance LC (Waters). For ultra performance LC-MS analysis, the mobile phases for positive electrospray ionization consisted of acetonitrile/water (50:50)/10 mm NH_4_OAc, pH5.0 (buffer A) and acetonitrile/water (99:1)/10 mm NH_4_OAc, pH5.0 (buffer B). The samples were separated on an ACQUITY ultra performance LC BEH Amide column (Waters, 2.1 mm × 100 mm, 1.8 μm) by gradient elution (50–99% buffer A in 1–4 min with a flow rate 400 μl/min), with a column temperature of 25 °C. Mass spectrometric conditions were set to spray voltage of 3.2 kV, sheath gas flow rate of 50 liters/min, auxiliary gas flow rate of 15 liters/min, and capillary temperature of 360 °C. Full scan MS spectra were acquired in the Orbitrap (*m*/*z* 50–250). The most intense ions were selected, and a 38eV higher energy collision dissociation with a 2 *m*/*z* isolation width was utilized. The signal to noise ratio was set to 3.0.

We determined the limit of detection using l-12C-cysteine (Sigma) standard solutions prepared in extraction buffer (50:50 methanol/water), and the limit of detection was 3.5 μg/ml. The standard curve was generated using l-12C-cysteine at six different concentrations (3.5–35 μg/ml), and 21 μg/ml of l-[1–13C]-cysteine was added into each standard solution as an internal control. The standard curve was generated using the peak areas ratio of l-C12-cysteine and l-[1–13C]-cysteine. The cysteine contents extracted from different strains were calculated using the standard linear equation, where *y* is the peak area ratio, and *x* is the concentration of l-12C-cysteine (45).

## Results

### 

#### 

##### P238L Mutation on RhlB Affects Its Interaction with PNPase but Not RNase E

**FIGURE 1.**
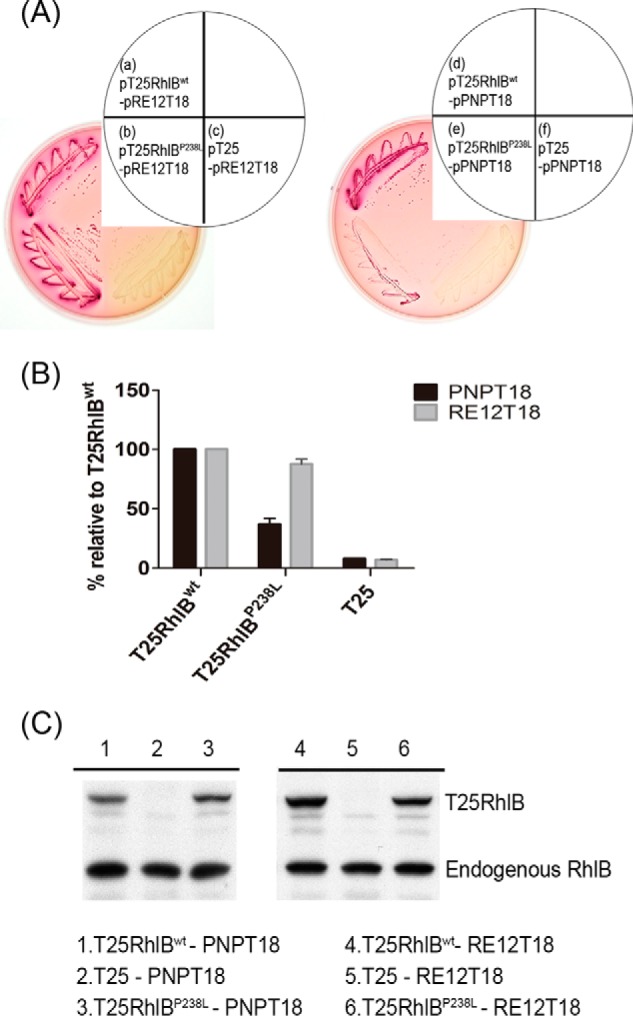
**RhlB P238L mutation affects interactions with PNPase but not RNase E.** *A*, DHP1 carrying the plasmids encoding different test proteins were plated on a MacConkey/maltose plate as indicated to visualize protein-protein interactions. The colonies carrying pT25RhlB with pRE12T18 (*panel a*) or pPNPT18 (*panel d*) were positive controls (8). The colonies carrying pT25 with pRE12T18 (*panel c*) or pPNPT18 (*panel f*) were negative controls. *B*, β-galactosidase assays were used to study the *in vivo* strength of protein-protein interactions. The interactions of PNPT18 (*black bar*) or RE12T18 (*gray bar*) with different T25RhlB constructs are shown. The reaction activities are given relative to the activity of T25RhlB^wt^, which was set as 100%. *C*, Western blot; total cell lysate of cells carrying the plasmids encoding different test proteins as indicated were resolved and detected using α-RhlB antibody.

It has previously been shown that PNPase and RhlB can interact independently of RNase E degradosome formation by bacterial two-hybrid and immunoprecipitation experiments (8, 9), but the importance of this interaction has remained unclear. To address this question, we isolated a mutant RhlB with impaired ability to interact with PNPase through a bacterial two-hybrid system. Random mutations were introduced into the *rhlB* gene by error prone PCR, and the resulting constructs were cloned into the pT25 plasmid (31). This pT25RhlB plasmid with randomly introduced mutations was cotransformed with wild-type pPNPT18 into the DHP1 strain for blue and white colony selection. T25RhlB with normal PNPT18-interacting ability induces CyaA activity, and its interaction with the promoter of the reporter gene *Lac*Z results in blue colonies on isopropyl β-d-thiogalactopyranoside/X-gal plates (31). We isolated plasmids from 314 white colonies of a total of 2000 on isopropyl β-d-thiogalactopyranoside/X-gal plates, indicative of cells potentially harboring a mutant RhlB with impaired PNPase interactions. After sequencing, we identified a mutation, P238L, located in the C-terminal region of RhlB. Because residues 194–421 of RhlB have previously been shown to interact with PNPase and with residues 684–784 of RNase E, RE12 (8), we further examined this RhlB^P238L^ mutant to see whether it interacts with RE12 like wild-type RhlB. The RhlB^P238L^ mutant interacted normally with RE12 (Fig. 1*A*, *panel a versus panel b*) but exhibited reduced interaction with PNPase compared with that of wild-type RhlB (Fig. 1*A*, *panel d versus panel e*). Consistently, the β-galactosidase activity of *E. coli* cells carrying the pT25RhlB^P238L^ mutant and pPNPT18 was less than that of pT25RhlB^wt^ and pPNPT18 (Fig. 1*B*) but similar between the strains carrying pT25RhlB^wt^ and pRE12T18 or pT25RhlB^P238L^ and pRE12T18. Western blots confirmed that the protein abundance of T25RhlB^P238L^ was comparable to that of T25RhlB^wt^ (Fig. 1*C*), confirming that the reduced color in the *E. coli* two-hybrid assay and the reduced β-galactosidase activity are due to weakened interaction between the RhlB^P238L^ mutant and PNPase. These data suggested that Pro-238 of RhlB is important for interactions with PNPase.

##### Mutation P238L Reduces the Association Rate of RhlB and PNPase

**FIGURE 2.**
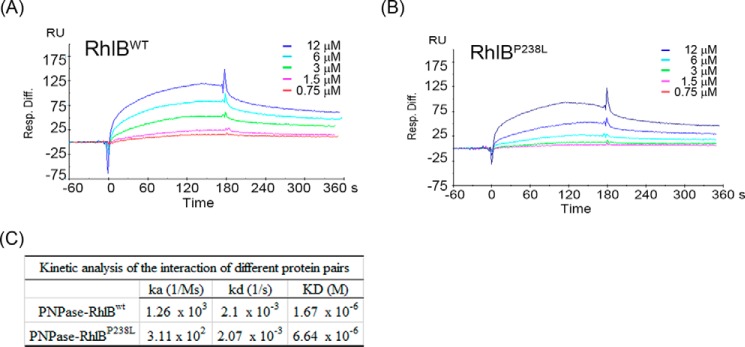
**Mutation P238L reduces the association rate of RhlB and PNPase.** *A* and *B*, kinetic data sets were collected for individual proteins (FlagRhlB^wt^ and FlagRhlB^P238L^) binding to a PNPase surface chip. Wild-type or P238L mutant RhlB proteins at various concentrations were injected over the PNPase surface. Individual injections were performed at least four times. *C*, the kinetic data for the association rate (*k_a_*) and dissociation rate (*k_d_*) constants determined in *A* and *B* were calculated by BIAcore evaluation software.

We next examined whether the association or dissociation rate constants of RhlB and PNPase were affected by the P238L mutation. The binding profiles of purified proteins FlagPNPase, FlagRhlB^wt^, and FlagRhlB^P238L^ were analyzed using BIAcore surface plasmon resonance (Fig. 2, *A* and *B*). We found that the association rate constant of RhlB^P238L^-PNPase (3.11 × 10^2^ m^−1^ s^−1^) is only 25% that of RhlB^wt^-PNPase (1.26 × 10^3^ m^−1^ s^−1^) (Fig. 2*C*). In contrast to the association rate constant that was affected by the P238L mutation, the dissociation rate constant remained similar for interactions between both the wild-type and P238L-mutated RhlB and PNPase (Fig. 2*C*). These results indicate that the P238L mutation decreases the binding affinity of RhlB with PNPase by reducing the rate of association.

##### RhlB P238L Mutant Retains Similar ATP-dependent Helicase Activity as RhlB Wild Type

**FIGURE 3.**
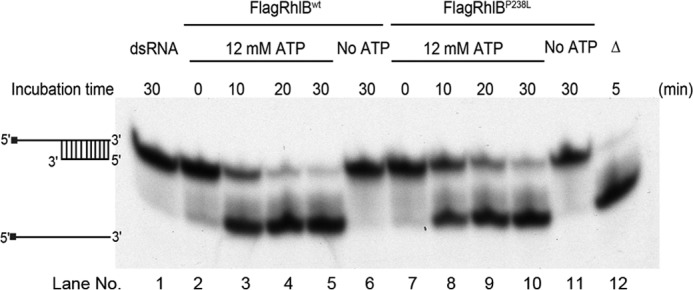
**RhlB P238L mutant retains similar ATP-dependent helicase activity as RhlB wild type.** The synthesized long (22-mer) RNA substrates were labeled at their 5′-ends with [γ-^32^P]ATP as indicated to the left of the blot. The *vertical lines* between long and short (11-mer) RNA substrates indicate the base-paired region. *Lane 1* contains the RNA duplex, whereas *lane 12* contains a heat-denatured control showing separated single-strand long RNA. ATP was added to samples containing FLAG-tagged protein and duplex RNA and incubated up to 30 min. *Lanes 2–5* and *7–10* show the RNA unwinding activities of FlagRhlB^wt^ and FlagRhlB^P238L^ in the presence of ATP over 30 min. *Lanes 6* and *11* show that FlagRhlB^wt^ and FlagRhlB^P238L^ has no RNA unwinding activity in the absence of ATP.

To clarify whether the P238L mutation on RhlB affects the unwinding activity of RhlB, we performed an RNA helicase activity assay. As shown in Fig. 3, FlagRhlB^wt^ and FlagRhlB^P238L^ were able to unwind duplex RNA (*lanes 2–5* and *7–10*, respectively), and these reactions were ATP-dependent (compare *lanes 2–5* to *lane 6* and *lanes 7–10* to *lane 11*). This result indicated that the P238L mutation on RhlB does not affect its ATP-dependent RNA unwinding activity.

##### RhlB P238L Mutation Alters mRNA Abundance of Cysteine Regulon Genes by Regulating the Degradation of cysB

**FIGURE 4.**
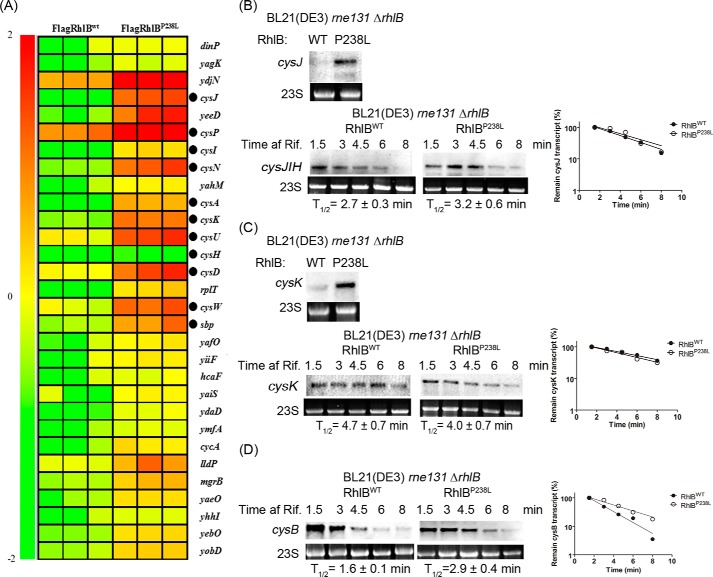
**RhlB P238L mutation alters mRNA abundance of cysteine regulon genes by regulating the degradation of *cysB*.** *A*, relative mRNA abundance was measured as described under “Experimental Procedures” and arranged according to the n-fold change (−2 < *n* < 2) between FlagRhlB^P238L^ and FlagRhlB^wt^. The microarray data show the top 30 genes (shown in three replicates), whose expression levels were altered by the expression of the P238L mutant RhlB. Gene names are shown to the *right* of the panel. A reference color bar on the *left* indicates the correlation between the observed color patterns and quantitative changes. *Closed circles* indicate genes belonging to the cysteine regulon. *B* and *C*, the steady-state level and the stability of *cysJIH* (*B*) and *cysK* (*C*) in BL21(DE3) *rne131* Δ*rhlB* containing FlagRhlB^wt^ or FlagRhlB^P238L^ were analyzed by Northern blot. *D*, the stability of the transcript *cysB* was analyzed and showed 1.8-fold stabilization upon expression of FlagRhlB^P238L^. The culture samples were collected at different time points after rifampicin treatment (*Time af Rif.*) as indicated. The half-life (*T*_½_) of each transcript was determined using the intensity of signals normalized to 23S rRNA, and the values are shown in a semi-logarithmic plot at the *right* of each panel.

Our previous *in vitro* study showed that RhlB helps PNPase degrade duplex RNA (8). To investigate the effects of RhlB and PNPase interactions independently of RNase E degradosome formation on mRNA abundance *in vivo*, we designed a microarray assay to examine changes in mRNA expression in cells harboring the RhlB C-terminal mutation P238L. Wild-type and mutant RhlB were introduced into an *E. coli* strain lacking the *rhlB* gene and carrying a truncated *rne* gene (BL21(DE3) *rne*131 Δ*rhlB*), with the *rne131* mutation encoding a truncated RNase E (residues 1–584) that prevents it from forming a degradosome complex with RhlB and PNPase (29); both wild-type and mutant RhlB were expressed at comparable levels (data not shown). The microarray data were analyzed as described under “Experimental Procedures.” Among the 220 significantly expressed genes, expression of FlagRhlB^P238L^ increased the abundance of the messages encoded by 69 genes (31.4%) more than 1.5-fold (Fig. 4*A*). Interestingly, 11 of the 69 genes belong to the cysteine regulon, strongly suggesting that the interaction of RhlB and PNPase is involved in regulating the expression of this set of genes, which is responsible for cysteine biosynthesis.

We next explored whether the altered expression of the cysteine regulon occurs at the transcriptional or post-transcriptional level. Northern blots revealed no significant differences in the stability of *cysJIH* and *cysK* transcripts in the strains expressing FlagRhlB^wt^ and FlagRhlB^P238L^ (Fig. 4, *B* and *C*). However, consistent with microarray data, the abundance of both *cysJIH* and *cysK* transcripts were increased in cells with a weakened RhlB-PNPase interaction (Fig. 4, *B* and *C*), indicating altered transcriptional control over these transcripts.

Taken together with the changes in multiple cysteine regulon genes and the fact that this control appeared to be occurring at the transcriptional level, we focused attention on the regulatory protein CysB. CysB is a dual transcription factor that is responsible for activating expression of the cysteine regulon with the exception of *cysE.* The attenuated RhlB-PNPase interaction resulted in stabilization of the *cysB* transcript ∼1.8-fold (Fig. 4*D*). These data indicate that RhlB-PNPase post-transcriptionally regulates the stability of *cysB* and controls the expression of the cysteine regulon.

##### RhlB-PNPase Regulates the Stability of cysB Independently of the RNA Degradosome

**FIGURE 5.**
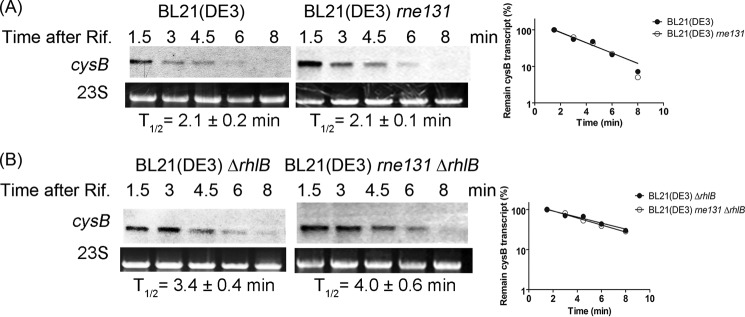
**RhlB-PNPase regulates the stability of *cysB* independently of the RNA degradosome.** Northern blot analyses were carried out over a time course following rifampicin treatment (*Time after Rif.*) to analyze the stability of *cysB* in different strains. BL21(DE3) and BL21(DE3) *rne131* are shown in *A*, and BL21(DE3) Δ*rhlB* and BL21(DE3) *rne131* Δ*rhlB* are shown in *B*. The half-life (*T*_½_) of *cysB* was determined using the intensity of signals normalized to 23S rRNA, and the semi-logarithmic plot is shown to the *right* of each panel.

To clarify the effect of RNA degradosome formation on the RhlB-PNPase target *cysB*, we compared the stability of *cysB* in the strain with full-length or C-terminally truncated RNase E and found that RNA degradosome formation does not affect the stability of *cysB* (Fig. 5*A*). We found that disruption of the interaction between RhlB and PNPase increases the stability of *cysB*. Thus, we wondered whether RhlB is required to regulate the degradation of *cysB*. The results showed that deletion of *rhlB* stabilized *cysB* more than 1.5-fold in the background of both full-length and C-terminal truncated RNase E (Fig. 5, *A versus B*). These results demonstrated that RhlB is required for regulation of the stability of *cysB* independently of RNA degradosome formation.

##### The Degradation of cysB Requires the Activity of RhlB, PNPase, and PcnB

**FIGURE 6.**
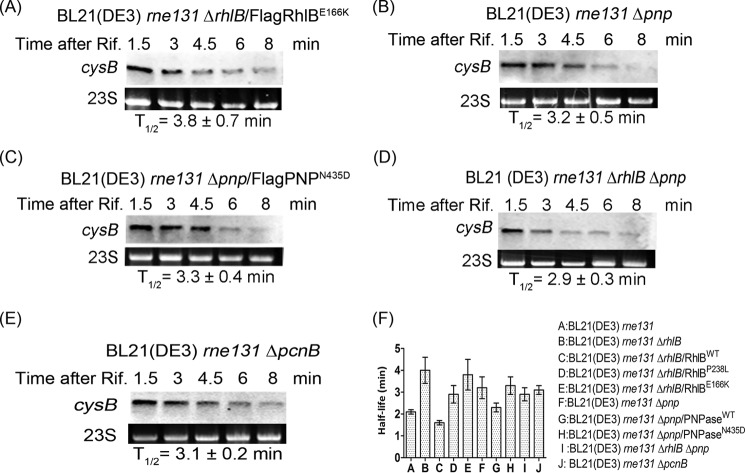
**The degradation of *cysB* requires the activity of RhlB, PNPase, and PcnB.** *A–E*, the stability of *cysB* in BL21(DE3) *rne131* Δ*rhlB* containing FlagRhlB^E166K^ (*A*), in BL21 *rne131* Δ*pnp* (*B*), in BL21 *rne131* Δ*pnp* containing FlagPNP^N435D^ (*C*), in BL21 *rne131* Δ*rhlB* Δ*pnp* (*D*), and in BL21 *rne131* Δ*pcnB* (*E*) were analyzed over a time course following rifampicin treatment (*Time after Rif.*) by Northern blot. The half-life (*T*_½_) of *cysB* was determined using the intensity of signals normalized to 23S rRNA. *F*, bar chart showing the mean half-life of *cysB* in different strains. *Error bars* represent standard errors.

Our results showed that interaction between RhlB and PNPase is required to regulate degradation of *cysB*, so we then set out to ascertain whether individual RhlB or PNPase activity is also required. The *cysB* stability was determined with DEAD box motif mutated RhlB^E166K^ (29), a PNPase deletion, or low phosphorolysis activity PNPase^N435D^ (30) (Fig. 6, *A–C*). The results showed that the stability of *cysB* was comparable in *rhlB* deletion and FlagRhlB^E166K^-expressing strains (Fig. 6*F*). Furthermore, the stability of *cysB* was comparable in the *pnp* deletion strain complemented with wild-type PNPase and in the parental BL21(DE3) *rne131* strain (Fig. 6*F*). Those results suggested that in addition to the protein-protein interaction, the activities of both RhlB and PNPase are required for *cysB* degradation. Combining *pnp* and *rhlB* deletions had no additional effect (Fig. 6*D*), thus demonstrating that PNPase and RhlB were regulating *cysB* degradation in the same pathway. Additionally, it has previously been shown that the Rho-independent terminator serves as a signal for polyadenylation by PcnB (46). The polyadenylated transcript has also been suggested to facilitate RNA degradation by PNPase (47). To examine the effect of polyadenylation on the degradation of *cysB*, we determined stability of *cysB* in the *pcnB* deletion background and found that the half-life of *cysB* is similar to that of the FlagRhlB^P238L^-expressing strain (Fig. 6*E versus right panel* of Fig. 4*B*). Consistent with our result that the RhlB-PNPase complex regulates the degradation of *cysB* in a 3′ to 5′ direction, PcnB also facilitates degradation of *cysB* at its 3′-end. Based on these results, under “Discussion,” we present a molecular mechanism for regulation of *cysB* mRNA degradation in a 3′ to 5′ direction that is influenced by RhlB, PNPase, and PcnB, independently of degradosome formation.

##### The Effect of Disrupting RhlB-PNPase Complex Formation in Controlling the Homeostatic Level of Cysteine

**FIGURE 7.**
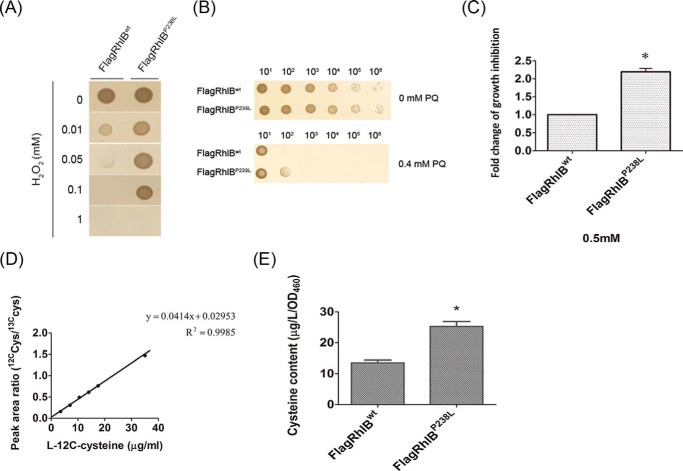
**The effect of disrupting RhlB-PNPase complex formation in controlling the homeostatic level of cysteine.** BL21(DE3) *rne131* Δ*rhlB* Δ*tnaA* Δ*cysE* containing pACYCDeut-CysE^M256I^ with pFlagRhlB^wt^ or pFlagRhlB^P238L^ were used for biological function analysis. *A* and *B*, anti-oxidative stress analysis was performed by spotting assays using different concentrations of H_2_O_2_ (*A*) or 0.4 mm PQ (*B*). *C*, growth inhibition was measured by comparing the cells treated with 0.5 mm cysteine and with H_2_O during early log phase (*OD*_460_ = 0.4). The relative change in cell density (*OD*_460_) caused by addition of cysteine was calculated as described under “Experimental Procedures.” The 2-fold change in growth inhibition relative to the strain expressing wild-type RhlB is shown. *D* and *E*, cysteine content was measured as described under “Experimental Procedures.” *D*, the standard curve of l-12C-cysteine with 21 μg/ml of l-[1–13C] cysteine as internal standard. The *x* axis shows the concentration of l-12C-cysteine (3.5–35 μg/ml). The *y* axis represents the LC-MS/MS signal ratio of l-12C-cysteine to l-[1–13C] cysteine. The equation and *R*^2^ of the standard curve is shown. *E*, metabolites were extracted from strains and applied to LC-MS/MS analysis as described in “Experimental Procedures.” * indicates *p* value of <0.05 as a statistically significant difference.

We have shown that interacting RhlB-PNPase regulates the stability of *cysB* and, further, that it modulates the expression of the cysteine regulon. Because cysteine is essential for many normal cellular functions, we examined the broader biological consequences of impaired interactions between RhlB and PNPase. Cysteine plays a major role in maintaining an intracellular reducing environment, thus protecting against oxidative stress (48). We treated *E. coli* carrying the wild-type or P238L mutant RhlB with the oxidative stress inducers H_2_O_2_ and PQ (an agent that diverts electrons from NADH or NADPH to molecular oxygen to generate a flux of superoxide (49)) and examined growth ability. We observed improved survival in the P238L mutant, suggesting improved oxidative stress resistance (Fig. 7, *A* and *B*).

Cysteine is also known to inhibit cell growth (50), so we also studied growth inhibition in wild-type and P238L mutants by adding 0.5 mm cysteine to the growth media, which we anticipated would amplify the growth inhibition induced by intracellular cysteine levels. We expected to observe reduced growth in our mutant with up-regulated cysteine regulon expression. As expected, cells expressing RhlB^P238L^ did exhibit 2-fold increased growth inhibition compared with the wild-type (Fig. 7*C*). To directly measure the cysteine content *in vivo*, we performed LC-MS/MS analysis with extracted metabolites from *E. coli* as described under “Experimental Procedures.” We determined the cysteine content by using the standard curve of l-12C-cysteine, which is generated using peak area ratios of l-C12-cysteine and l-[1–13C]-cysteine (Fig. 7*D*). Although the intracellular content of cysteine in cells expressing FlagRhlB^wt^ was 13.5 μg/liter/*OD*_460_, cells bearing the FlagRhlB^P238L^ mutant had an increased cysteine content of 25.3 μg/liter/*OD*_460_ (Fig. 7*E*), indicating that the P238L mutation resulted not only in increased cysteine regulon expression but, ultimately, increased intracellular cysteine content.

## Discussion

RhlB has been shown *in vitro* to facilitate PNPase degradation of double-stranded RNA (8). Here, we report that the P238L mutation attenuates the interaction of RhlB and PNPase and is important in regulating the stability of *cysB*, which further modulates the homeostatic levels of cysteine. This is the first report linking RhlB-PNPase to cysteine biosynthesis and showing that the protein-protein interaction is necessary for this regulation. In addition, the regulation of *cysB* by RhlB-PNPase is independent of the RNA degradosome (Fig. 5). In contrast, *rpsT* and the repetitive extragenic palindrome-containing transcript *malEF* has been shown to be regulated by RhlB and PNPase, but this regulation requires RNA degradosome formation (13). Previous reports have shown that the RhlB-PNPase complex can form independently of the RNase E-based degradosome (9), but we show that this degradosome-independent interaction plays a distinct role in controlling gene expression in cells.

**FIGURE 8.**
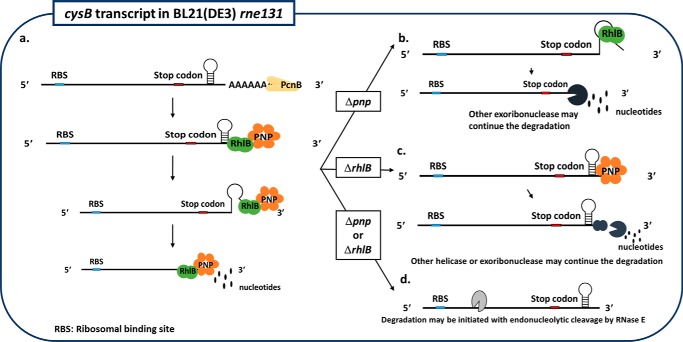
**Regulation of 3′ to 5′ degradation of *cysB* transcript in absence of degradosome formation.** *a*, *cysB* transcript contains a ribosomal binding site (*RBS*) at its 5′-end with a Rho-independent terminator in the form of a stem loop at its 3′-end downstream of the stop codon, which inhibits the 3′ to 5′ exoribonuclease activity of PNPase. Poly(A) polymerase (PcnB) adds additional A residues to the transcript to facilitate the degradation of *cysB*. Furthermore, RhlB binds to the 3′-end of the *cysB* transcript and unwinds the secondary structure, thereby assisting in the degradation of *cysB* by PNPase independently of RNase E-degradosome formation. *b*, in the absence of PNPase, the unwound *cysB* can be further degraded by other exoribonucleases, for example, ribonuclease II. *c*, in the absence of RhlB, other helicases or exoribonucleases may replace the function of the RhlB-PNPase complex. *d*, in the absence of PNPase or RhlB, degradation of *cysB* may begin with endonucleolytic cleavage by RNase E.

We show that the interaction of RhlB-PNPase regulates the stability of the *cysB* transcript and the abundance of *cys* transcripts. Weakening the protein interaction of RhlB-PNPase can thus stabilize *cysB*, increase CysB protein levels, and ultimately lead to the increased abundance of *cys* transcripts that we observed. However, CysB itself auto-regulates the expression of *cysB* and, as a result, the weakly interacting RhlB-PNPase increased the steady-state level of *cysB* only 1.3-fold, although this small increase of *cysB* mRNA had a significant impact on controlling the cysteine regulon. This regulation is independent of its association with the RNA degradosome but dependent on PNPase (Fig. 5–8). In addition, PcnB also facilitates the degradation of *cysB* by polyadenylation (Fig. 6*E*). However, FlagRhlB^P238L^-expressing cells with normal PcnB activity can sufficiently stabilize *cysB* mRNA, suggesting that PNPase requires RhlB association to unwind the secondary structure and that polyadenylation may enhance the binding of PNPase. We suggest a molecular mechanism of *cysB* degradation in a 3′ to 5′ direction independently of degradosome formation (Fig. 8). As shown in Fig. 8*a*, the PcnB adds polyadenosine at the 3′-end of *cysB*, which facilitates the binding of the degradosome-independent RhlB-PNPase complex. RhlB further unwinds the strong secondary structure downstream of the stop codon (which cannot be degraded by PNPase alone), thereby allowing the unwound transcript to be degraded by PNPase. Because CysB plays an important role in controlling the homeostasis of cysteine, the expression levels of its transcript must be tightly controlled. In the absence of PNPase (Fig. 8*b*) or RhlB (Fig. 8*c*), the degradation of *cysB* may be compensated by other helicases and exoribonucleases. This possibility is supported by previous studies that show *pnp* deletion increases the expression level and activity of ribonuclease II (51) and that the expression level of SrmB was increased in an *rhlB* deletion strain (52). The degradation of *cysB* may also begin with endonucleolytic cleavage by RNase E and may be faster than 3′ exonucleolytic attack when either PNPase or RhlB is absent (Fig. 8*d*). Because our experiments were done with the *rne131* strain that lacks degradosome formation, we cannot exclude the possibility that additional pathway(s) involved in *cysB* degradation can occur in a strain having RNase E-degradosome formation.

Recently, the exosome of the bacterium *Deinococcus radiodurans* was reported as an Rsr-Y RNA-PNPase RNP complex, wherein Rsr and PNPase form a complex with Y RNA to facilitate the degradation of double-stranded RNA (53). However, neither Rsr protein nor Y RNA homologs exist in *E. coli*. In *E. coli*, purified RhlB and PNPase interact directly without assistance (9). Here, we found that disruption of the interaction of RhlB and PNPase increased the intracellular cysteine content and enhanced anti-oxidative resistance (Fig. 7). Hence, our study suggests a role for the RhlB-PNPase complex in modulating oxidative stress responses by controlling cysteine synthesis through regulating the stability of *cysB*. Previous studies have reported that *pnp* deletion strains exhibit heightened resistance to the oxidative stress agents H_2_O_2_ and PQ (54, 55). Under similar oxidative stress conditions, other studies have also shown that the activity and protein level of hPNPase are reduced in HeLa cells (56) and that PNPase protein abundance is reduced in bacteria (57). Moreover, it has been shown in *E. coli* that binding of metabolites such as citrate inhibits both the degradation and polymerization activities of PNPase (58). Whether the binding of metabolites can also regulate the interaction of RhlB and PNPase remains to be studied.

## Author Contributions

N.-T. C. designed, performed, and analyzed the experiments shown in Figs. 1–3. R. G. performed and analyzed the experiments shown in Fig. 4*A*. Y.-T. T. designed, performed, and analyzed the experiments shown in Figs. 4 (*B–D*) and 5–7. N.-T. C., R. G., and Y.-T. T. contributed to the writing, drafting, and preparation of the manuscript. S. L.-C. contributed to the overall study design and interpretation of the results during all phases and edited the final manuscript. All authors reviewed the results and approved the final version of the manuscript.

## Footnotes

3 The abbreviations used are:

PNPase: polynucleotide phosphorylase
RhlB: RNA helicase B
RNase E: ribonuclease E
PQ: paraquat.

## References

[1] Blum E., Py B., Carpousis A. J., and Higgins C. F. (1997) Polyphosphate kinase is a component of the *Escherichia coli* RNA degradosome. Mol. Microbiol. 26, 387–398 9383162 10.1046/j.1365-2958.1997.5901947.x

[2] Morita T., Maki K., and Aiba H. (2005) RNase E-based ribonucleoprotein complexes: mechanical basis of mRNA destabilization mediated by bacterial noncoding RNAs. Genes Dev. 19, 2176–2186 16166379 10.1101/gad.1330405 PMC1221888

[3] Gao J., Lee K., Zhao M., Qiu J., Zhan X., Saxena A., Moore C. J., Cohen S. N., and Georgiou G. (2006) Differential modulation of *E. coli* mRNA abundance by inhibitory proteins that alter the composition of the degradosome. Mol. Microbiol. 61, 394–406 16771842 10.1111/j.1365-2958.2006.05246.x

[4] Singh D., Chang S. J., Lin P. H., Averina O. V., Kaberdin V. R., and Lin-Chao S. (2009) Regulation of ribonuclease E activity by the L4 ribosomal protein of *Escherichia coli*. Proc. Natl. Acad. Sci. U.S.A. 106, 864–869 19144914 10.1073/pnas.0810205106 PMC2626609

[5] Carpousis A. J., Van Houwe G., Ehretsmann C., and Krisch H. M. (1994) Copurification of *E. coli* RNAase E and PNPase: evidence for a specific association between two enzymes important in RNA processing and degradation. Cell 76, 889–900 7510217 10.1016/0092-8674(94)90363-8

[6] Miczak A., Kaberdin V. R., Wei C. L., and Lin-Chao S. (1996) Proteins associated with RNase E in a multicomponent ribonucleolytic complex. Proc. Natl. Acad. Sci. U.S.A. 93, 3865–3869 8632981 10.1073/pnas.93.9.3865 PMC39450

[7] Py B., Higgins C. F., Krisch H. M., and Carpousis A. J. (1996) A DEAD-box RNA helicase in the *Escherichia coli* RNA degradosome. Nature 381, 169–172 8610017 10.1038/381169a0

[8] Liou G. G., Chang H. Y., Lin C. S., and Lin-Chao S. (2002) DEAD box RhlB RNA helicase physically associates with exoribonuclease PNPase to degrade double-stranded RNA independent of the degradosome-assembling region of RNase E. J. Biol. Chem. 277, 41157–41162 12181321 10.1074/jbc.M206618200

[9] Lin P. H., and Lin-Chao S. (2005) RhlB helicase rather than enolase is the β-subunit of the *Escherichia coli* polynucleotide phosphorylase (PNPase)-exoribonucleolytic complex. Proc. Natl. Acad. Sci. U.S.A. 102, 16590–16595 16275923 10.1073/pnas.0500994102 PMC1277965

[10] Wong A. G., McBurney K. L., Thompson K. J., Stickney L. M., and Mackie G. A. (2013) S1 and KH domains of polynucleotide phosphorylase determine the efficiency of RNA binding and autoregulation. J. Bacteriol. 195, 2021–2031 23457244 10.1128/JB.00062-13 PMC3624587

[11] Shi Z., Yang W. Z., Lin-Chao S., Chak K. F., and Yuan H. S. (2008) Crystal structure of *Escherichia coli* PNPase: central channel residues are involved in processive RNA degradation. RNA 14, 2361–2371 18812438 10.1261/rna.1244308 PMC2578853

[12] Spickler C., and Mackie G. A. (2000) Action of RNase II and polynucleotide phosphorylase against RNAs containing stem-loops of defined structure. J. Bacteriol. 182, 2422–2427 10762241 10.1128/jb.182.9.2422-2427.2000 PMC111303

[13] Coburn G. A., Miao X., Briant D. J., and Mackie G. A. (1999) Reconstitution of a minimal RNA degradosome demonstrates functional coordination between a 3′ exonuclease and a DEAD-box RNA helicase. Genes Dev. 13, 2594–2603 10521403 10.1101/gad.13.19.2594 PMC317069

[14] Lin-Chao S., Chiou N. T., and Schuster G. (2007) The PNPase, exosome and RNA helicases as the building components of evolutionarily-conserved RNA degradation machines. J. Biomed Sci. 14, 523–532 17514363 10.1007/s11373-007-9178-y

[15] Januszyk K., and Lima C. D. (2011) Structural components and architectures of RNA exosomes. Adv. Exp. Med. Biol. 702, 9–28 21713674 10.1007/978-1-4419-7841-7_2

[16] van Hoof A., Lennertz P., and Parker R. (2000) Yeast exosome mutants accumulate 3′-extended polyadenylated forms of U4 small nuclear RNA and small nucleolar RNAs. Mol. Cell. Biol. 20, 441–452 10611222 10.1128/mcb.20.2.441-452.2000 PMC85098

[17] Chen C. Y., Gherzi R., Ong S. E., Chan E. L., Raijmakers R., Pruijn G. J., Stoecklin G., Moroni C., Mann M., and Karin M. (2001) AU binding proteins recruit the exosome to degrade ARE-containing mRNAs. Cell 107, 451–464 11719186 10.1016/s0092-8674(01)00578-5

[18] Tran H., Schilling M., Wirbelauer C., Hess D., and Nagamine Y. (2004) Facilitation of mRNA deadenylation and decay by the exosome-bound, DExH protein RHAU. Mol. Cell 13, 101–111 14731398 10.1016/s1097-2765(03)00481-7

[19] LaCava J., Houseley J., Saveanu C., Petfalski E., Thompson E., Jacquier A., and Tollervey D. (2005) RNA degradation by the exosome is promoted by a nuclear polyadenylation complex. Cell 121, 713–724 15935758 10.1016/j.cell.2005.04.029

[20] Wyers F., Rougemaille M., Badis G., Rousselle J. C., Dufour M. E., Boulay J., Régnault B., Devaux F., Namane A., Séraphin B., Libri D., and Jacquier A. (2005) Cryptic pol II transcripts are degraded by a nuclear quality control pathway involving a new poly(A) polymerase. Cell 121, 725–737 15935759 10.1016/j.cell.2005.04.030

[21] Szczesny R. J., Borowski L. S., Brzezniak L. K., Dmochowska A., Gewartowski K., Bartnik E., and Stepien P. P. (2010) Human mitochondrial RNA turnover caught in flagranti: involvement of hSuv3p helicase in RNA surveillance. Nucleic Acids Res. 38, 279–298 19864255 10.1093/nar/gkp903 PMC2800237

[22] Lubas M., Christensen M. S., Kristiansen M. S., Domanski M., Falkenby L. G., Lykke-Andersen S., Andersen J. S., Dziembowski A., and Jensen T. H. (2011) Interaction profiling identifies the human nuclear exosome targeting complex. Mol. Cell 43, 624–637 21855801 10.1016/j.molcel.2011.06.028

[23] Borowski L. S., Dziembowski A., Hejnowicz M. S., Stepien P. P., and Szczesny R. J. (2013) Human mitochondrial RNA decay mediated by PNPase-hSuv3 complex takes place in distinct foci. Nucleic Acids Res. 41, 1223–1240 23221631 10.1093/nar/gks1130 PMC3553951

[24] Barrett E. L., and Chang G. W. (1979) Cysteine auxotrophs of *Salmonella typhimurium* which grow without cysteine in a hydrogen/carbon dioxide atmosphere. J. Gen. Microbiol. 115, 513–516 393803 10.1099/00221287-115-2-513

[25] Joyce A. R., Reed J. L., White A., Edwards R., Osterman A., Baba T., Mori H., Lesely S. A., Palsson B. Ø., and Agarwalla S. (2006) Experimental and computational assessment of conditionally essential genes in *Escherichia coli*. J. Bacteriol. 188, 8259–8271 17012394 10.1128/JB.00740-06 PMC1698209

[26] Hryniewicz M. M., and Kredich N. M. (1994) Stoichiometry of binding of CysB to the cysJIH, cysK, and cysP promoter regions of *Salmonella typhimurium*. J. Bacteriol. 176, 3673–3682 8206845 10.1128/jb.176.12.3673-3682.1994 PMC205556

[27] Bernstein J. A., Lin P. H., Cohen S. N., and Lin-Chao S. (2004) Global analysis of *Escherichia coli* RNA degradosome function using DNA microarrays. Proc. Natl. Acad. Sci. U.S.A. 101, 2758–2763 14981237 10.1073/pnas.0308747101 PMC365694

[28] Datsenko K. A., and Wanner B. L. (2000) One-step inactivation of chromosomal genes in *Escherichia coli* K-12 using PCR products. Proc. Natl. Acad. Sci. U.S.A. 97, 6640–6645 10829079 10.1073/pnas.120163297 PMC18686

[29] Vanzo N. F., Li Y. S., Py B., Blum E., Higgins C. F., Raynal L. C., Krisch H. M., and Carpousis A. J. (1998) Ribonuclease E organizes the protein interactions in the *Escherichia coli* RNA degradosome. Genes Dev. 12, 2770–2781 9732274 10.1101/gad.12.17.2770 PMC317140

[30] Jarrige A., Bréchemier-Baey D., Mathy N., Duché O., and Portier C. (2002) Mutational analysis of polynucleotide phosphorylase from *Escherichia coli*. J. Mol. Biol. 321, 397–409 12162954 10.1016/s0022-2836(02)00645-9

[31] Karimova G., Pidoux J., Ullmann A., and Ladant D. (1998) A bacterial two-hybrid system based on a reconstituted signal transduction pathway. Proc. Natl. Acad. Sci. U.S.A. 95, 5752–5756 9576956 10.1073/pnas.95.10.5752 PMC20451

[32] Kido M., Yamanaka K., Mitani T., Niki H., Ogura T., and Hiraga S. (1996) RNase E polypeptides lacking a carboxyl-terminal half suppress a mukB mutation in *Escherichia coli*. J. Bacteriol. 178, 3917–3925 8682798 10.1128/jb.178.13.3917-3925.1996 PMC232654

[33] Lopez P. J., Marchand I., Joyce S. A., and Dreyfus M. (1999) The C-terminal half of RNase E, which organizes the *Escherichia coli* degradosome, participates in mRNA degradation but not rRNA processing *in vivo*. Mol. Microbiol. 33, 188–199 10411735 10.1046/j.1365-2958.1999.01465.x

[34] Schena M., Shalon D., Davis R. W., and Brown P. O. (1995) Quantitative monitoring of gene expression patterns with a complementary DNA microarray. Science 270, 467–470 7569999 10.1126/science.270.5235.467

[35] Bernstein J. A., Khodursky A. B., Lin P. H., Lin-Chao S., and Cohen S. N. (2002) Global analysis of mRNA decay and abundance in *Escherichia coli* at single-gene resolution using two-color fluorescent DNA microarrays. Proc. Natl. Acad. Sci. U.S.A. 99, 9697–9702 12119387 10.1073/pnas.112318199 PMC124983

[36] Lin-Chao S., and Cohen S. N. (1991) The rate of processing and degradation of antisense RNAI regulates the replication of ColE1-type plasmids *in vivo*. Cell 65, 1233–1242 1712252 10.1016/0092-8674(91)90018-t

[37] Holtke H. J., Ankenbauer W., Muhlegger K., Rein R., Sagner G., Seibl R., and Walter T. (1995) The digoxigenin (DIG) system for non-radioactive labelling and detection of nucleic acids: an overview. Cell Mol. Biol. (Noisy-le-grand) 41, 883–905 8595368

[38] Ostrowski J., and Kredich N. M. (1991) Negative autoregulation of *cysB* in *Salmonella typhimurium*: *in vitro* interactions of CysB protein with the cysB promoter. J. Bacteriol. 173, 2212–2218 1706701 10.1128/jb.173.7.2212-2218.1991 PMC207769

[39] Denk D., and Böck A. (1987) l-Cysteine biosynthesis in *Escherichia coli*: nucleotide sequence and expression of the serine acetyltransferase (cysE) gene from the wild-type and a cysteine-excreting mutant. J. Gen. Microbiol. 133, 515–525 3309158 10.1099/00221287-133-3-515

[40] Kredich N. M., and Tomkins G. M. (1966) The enzymic synthesis of l-cysteine in *Escherichia coli* and *Salmonella typhimurium*. J. Biol. Chem. 241, 4955–4965 5332668

[41] Yamada S., Awano N., Inubushi K., Maeda E., Nakamori S., Nishino K., Yamaguchi A., and Takagi H. (2006) Effect of drug transporter genes on cysteine export and overproduction in *Escherichia coli*. Appl. Environ. Microbiol. 72, 4735–4742 16820466 10.1128/AEM.02507-05 PMC1489377

[42] Nakamori S., Kobayashi S. I., Kobayashi C., and Takagi H. (1998) Overproduction of l-cysteine and l-cystine by *Escherichia coli* strains with a genetically altered serine acetyltransferase. Appl. Environ. Microbiol. 64, 1607–1611 9572924 10.1128/aem.64.5.1607-1611.1998 PMC106203

[43] Takagi H., Awano N., Kobayashi S., Noji M., Saito K., and Nakamori S. (1999) Overproduction of l-cysteine and l-cystine by expression of genes for feedback inhibition-insensitive serine acetyltransferase from *Arabidopsis thaliana* in *Escherichia coli*. FEMS Microbiol. Lett. 179, 453–459 10518750 10.1111/j.1574-6968.1999.tb08762.x

[44] Lu W., Kimball E., and Rabinowitz J. D. (2006) A high-performance liquid chromatography-tandem mass spectrometry method for quantitation of nitrogen-containing intracellular metabolites. J. Am. Soc. Mass. Spectrom. 17, 37–50 16352439 10.1016/j.jasms.2005.09.001

[45] Shuford C. M., Poteat M. D., Buchwalter D. B., and Muddiman D. C. (2012) Absolute quantification of free glutathione and cysteine in aquatic insects using isotope dilution and selected reaction monitoring. Anal. Bioanal. Chem. 402, 357–366 21956263 10.1007/s00216-011-5416-2

[46] Mohanty B. K., and Kushner S. R. (2006) The majority of *Escherichia coli* mRNAs undergo post-transcriptional modification in exponentially growing cells. Nucleic Acids Res. 34, 5695–5704 17040898 10.1093/nar/gkl684 PMC1636475

[47] Xu F., and Cohen S. N. (1995) RNA degradation in *Escherichia coli* regulated by 3′ adenylation and 5′ phosphorylation. Nature 374, 180–183 7533264 10.1038/374180a0

[48] Turnbull A. L., and Surette M. G. (2010) Cysteine biosynthesis, oxidative stress and antibiotic resistance in *Salmonella typhimurium*. Res. Microbiol. 161, 643–650 20600858 10.1016/j.resmic.2010.06.004

[49] Lascano R., Muñoz N., Robert G., Rodriguez M., Melchiorre M., Trippi V., and Quero G. (2012) Paraquat: an oxidative stress inducer. in Herbicides: Properties, Synthesis and Control of Weeds (Hasaneen M. N., ed), pp. 135–148, InTech, Rijeka, Croatia

[50] Kari C., Nagy Z., Kovács P., and Hernádi F. (1971) Mechanism of the growth inhibitory effect of cysteine on *Escherichia coli*. J. Gen. Microbiol. 68, 349–356 4944587 10.1099/00221287-68-3-349

[51] Zilhão R., Cairrão F., Régnier P., and Arraiano C. M. (1996) PNPase modulates RNase II expression in *Escherichia coli*: implications for mRNA decay and cell metabolism. Mol. Microbiol. 20, 1033–1042 8809756 10.1111/j.1365-2958.1996.tb02544.x

[52] Zhou L., Zhang A. B., Wang R., Marcotte E. M., and Vogel C. (2013) The proteomic response to mutants of the *Escherichia coli* RNA degradosome. Mol. Biosyst. 9, 750–757 23403814 10.1039/c3mb25513a PMC3709862

[53] Chen X., Taylor D. W., Fowler C. C., Galan J. E., Wang H. W., and Wolin S. L. (2013) An RNA degradation machine sculpted by Ro autoantigen and noncoding RNA. Cell 153, 166–177 23540697 10.1016/j.cell.2013.02.037 PMC3646564

[54] Hayakawa H., Kuwano M., and Sekiguchi M. (2001) Specific binding of 8-oxoguanine-containing RNA to polynucleotide phosphorylase protein. Biochemistry 40, 9977–9982 11502194 10.1021/bi010595q

[55] Henry A., Shanks J., van Hoof A., and Rosenzweig J. A. (2012) The *Yersinia pseudotuberculosis* degradosome is required for oxidative stress, while its PNPase subunit plays a degradosome-independent role in cold growth. FEMS Microbiol. Lett. 336, 139–147 23082859 10.1111/j.1574-6968.12000.x PMC5832447

[56] Hayakawa H., and Sekiguchi M. (2006) Human polynucleotide phosphorylase protein in response to oxidative stress. Biochemistry 45, 6749–6755 16716086 10.1021/bi052585l

[57] Xiao M., Xu P., Zhao J., Wang Z., Zuo F., Zhang J., Ren F., Li P., Chen S., and Ma H. (2011) Oxidative stress-related responses of *Bifidobacterium longum* subsp. longum BBMN68 at the proteomic level after exposure to oxygen. Microbiology 157, 1573–1588 21349974 10.1099/mic.0.044297-0

[58] Nurmohamed S., Vincent H. A., Titman C. M., Chandran V., Pears M. R., Du D., Griffin J. L., Callaghan A. J., and Luisi B. F. (2011) Polynucleotide phosphorylase activity may be modulated by metabolites in *Escherichia coli*. J. Biol. Chem. 286, 14315–14323 21324911 10.1074/jbc.M110.200741 PMC3077632

[59] Studier F. W., and Moffatt B. A. (1986) Use of bacteriophage T7 RNA polymerase to direct selective high-level expression of cloned genes. J. Mol. Biol. 189, 113–130 3537305 10.1016/0022-2836(86)90385-2

